# Environmental conditions and Puumala virus transmission in Belgium

**DOI:** 10.1186/1476-072X-6-55

**Published:** 2007-12-14

**Authors:** Catherine Linard, Katrien Tersago, Herwig Leirs, Eric F Lambin

**Affiliations:** 1 Department of Geography, Université Catholique de Louvain, Place Pasteur 3, B-1348 Louvain-la-Neuve, Belgium; 2 Research group of Evolutionary Biology, University of Antwerp, B-2020 Antwerp, Belgium; 3 Danish Pest Infestation Laboratory, University of Aarhus, 2800 Kongens Lyngby, Denmark

## Abstract

**Background:**

Non-vector-borne zoonoses such as Puumala hantavirus (PUUV) can be transmitted directly, by physical contact between infected and susceptible hosts, or indirectly, with the environment as an intermediate. The objective of this study is to better understand the causal link between environmental features and PUUV prevalence in bank vole population in Belgium, and hence with transmission risk to humans. Our hypothesis was that environmental conditions controlling the direct and indirect transmission paths differ, such that the risk of transmission to humans is not only determined by host abundance. We explored the relationship between, on one hand, environmental variables and, on the other hand, host abundance, PUUV prevalence in the host, and human cases of nephropathia epidemica (NE). Statistical analyses were carried out on 17 field sites situated in Belgian broadleaf forests.

**Results:**

Linear regressions showed that landscape attributes, particularly landscape configuration, influence the abundance of hosts in broadleaf forests. Based on logistic regressions, we show that PUUV prevalence among bank voles is more linked to variables favouring the survival of the virus in the environment, and thus the indirect transmission: low winter temperatures are strongly linked to prevalence among bank voles, and high soil moisture is linked to the number of NE cases among humans. The transmission risk to humans therefore depends on the efficiency of the indirect transmission path. Human risk behaviours, such as the propensity for people to go in forest areas that best support the virus, also influence the number of human cases.

**Conclusion:**

The transmission risk to humans of non-vector-borne zoonoses such as PUUV depends on a combination of various environmental factors. To understand the complex causal pathways between the environment and disease risk, one should distinguish between environmental factors related to the abundance of hosts such as land-surface attributes, landscape configuration, and climate – i.e., host ecology, – and environmental factors related to PUUV prevalence, mainly winter temperatures and soil moisture – i.e., virus ecology. Beyond a threshold abundance of hosts, environmental factors favouring the indirect transmission path (soil and climate) can better predict the number of NE cases among humans than factors influencing the abundance of hosts.

## Background

Ecosystem changes coupled with climatic change are thought to increase the probability of emergence or re-emergence of various infectious diseases, and particularly vector-borne and zoonotic diseases. Environmental conditions influence the geographic distribution of disease risk or incidence by controlling the spatial distribution of vectors and hosts, according to their habitat preferences, and by influencing the contact rate between vectors, hosts and humans [1,2].

Non-vector-borne zoonoses do not always require direct physical contact between infected and susceptible hosts for transmission to take place. Actually, the transmission can also be indirect, for example in aerosol form, with the environment as intermediate [3,4]. The environmental conditions that influence the direct and indirect transmission paths of non-vector-borne zoonoses may be different. For the direct transmission path, which involves direct contact between infected and susceptible hosts, environmental features such as land-surface attributes (surface and vegetation characteristics detected by remote sensing) or landscape configuration variables (spatial organisation of the elements composing the landscape) [5] influence the host population abundance according to its habitat ecology and its dispersal in the landscape [6-8]. The abundance of hosts is generally considered as the major factor contributing to disease transmission risk, by increasing contacts with humans. The indirect transmission path, dominated by the availability of infectious agents in the environment, depends not just on the abundance of infected hosts but also on environmental conditions that influence the survival of the virus in the natural environment, such as climate and soil characteristics. The indirect transmission favours both the virus transmission among the reservoir host population and the transmission to humans. The transmission to humans also varies according to human risk behaviours and their associated land uses. Some behaviours are associated with a close proximity to host habitat and thus increase the likelihood of human-host contacts. Other land uses bring humans into contact with areas that best support the virus.

The distinction between the direct and indirect transmission pathways applies to Puumala virus (PUUV), a hantavirus that causes a mild form of haemorrhagic fever with renal syndrome (HFRS) in western and northern Europe: nephropathia epidemica (NE) [9]. PUUV is predominantly associated with the bank vole (*Myodes glareolus*) [10-12] that, particularly in Western Europe, lives in a specific habitat mainly composed of broadleaf forests with dense undergrowth [11,13]. Bank voles are not evenly distributed in these forests as both structural factors and the quality of forest patches strongly influence the habitat suitability for bank voles [6-8,14]. Components of the landscape structure such as patch size, fragmentation and isolation influence the suitability of habitats for bank voles, their dispersal and the chance for metapopulations to survive [6-8]. Climatic conditions can also influence populations of rodents, by controlling vegetation growth, snow cover or food supply – e.g., via masting events during years of intense seed production of oak and beech induced by high summer temperatures [9,15]. The importance of the indirect mode of transmission and the prolonged survival of PUUV outside the rodent host was first highlighted by mathematical modelling [3] and then proven in cell culture [4]. The virus shed in urine and faeces of bank voles can remain infectious for 5–11 days in cell culture at room temperature [4]. Climate conditions and soil characteristics are thought to influence the survival of viruses in the soil litter [16]. It was shown for example that temperature and moisture influence the longevity of PUUV particles outside the host [4]. The abundance of infected bank voles has been frequently associated with wet habitats [17-19]. However, the risk of human infection could be greater when excretions become dry ([20] for the related Sin Nombre virus). Additional factors that favour the persistence of the virus in the natural environment, such as physical and chemical characteristics of the soil, are not well known [4,21]. The spatial distribution of PUUV infection among rodents generally covers boreal and temperate regions, suggesting that the environmental conditions of these regions are more appropriate for prolonged survival of PUUV [4]. However, the relative importance of the direct and indirect transmission route of PUUV in western Europe is not known.

The objective of this study is to better understand the causal link between environmental features and PUUV prevalence in bank vole population in Belgium, and hence with transmission risk to humans. We explored the relationship between environmental variables and host abundance, PUUV prevalence in the host and human cases of NE. Our hypothesis is that the impact of environmental attributes on non-vector-borne zoonoses cannot be reduced to its link with the spatial distribution of hosts, as a high abundance of hosts is not sufficient for transmission to occur. The indirect transmission path is expected to increase the prevalence in hosts and the transmission risk to humans. We expect that, while land-surface attributes and configuration influence the abundance of hosts (via host ecology), climate and soil conditions influence the prevalence of PUUV in hosts and humans, by favouring the indirect transmission path (via virus ecology). The relationship between human risk and the environmental factors that favour indirect transmission can be non-linear as the human prevalence depends on the prevalence in the host, and both are influenced by the indirect transmission. This may lead to a multiplicative effect.

The relationship between environmental features and the incidence of infectious diseases, particularly rodent-borne zoonoses, has already been investigated, e.g. using remote sensing to predict viral prevalence in hosts [22] or human incidence [23]. Few studies however have applied remote sensing to study the epidemiology of mammal reservoirs [24]. Several studies analysed spatial dynamics of diseases related to land-surface attributes, land cover and land use [22,25,19]. Landscape configuration was not often taken into account, even though its potential impact on infectious disease incidence was highlighted [5,26]. For examples of studies on rodent-borne diseases that consider the landscape configuration, see [7] and [20].

## Materials and methods

### Study zone

**Figure 1:**
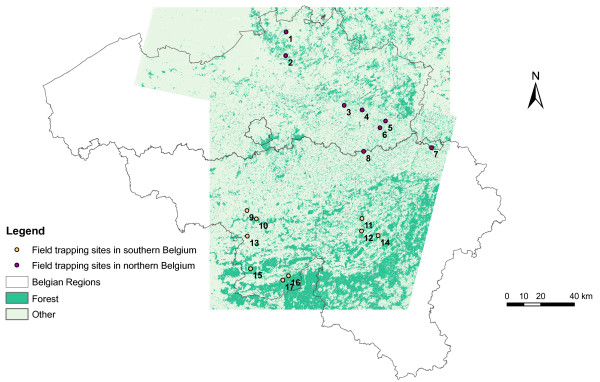
Study area: localization of field trapping sites and Landsat images.

Data were collected in 17 sites, among which 8 are situated in the northern part of Belgium (where NE incidence is low) and 9 in the southern part of Belgium (where NE human cases are concentrated) (Figure 1). All sites are located in mixed oak and beech forests. The minimum distance between 2 sites is 4.3 km, which is much larger than the dispersal distance of bank voles (estimated to be 500 meters in patchy landscapes [6]) and thus avoids spatial dependence between sites.

### Data collection

#### Field trapping data

Data were collected during two consecutive years. In northern Belgium, we trapped in 8 sites in August or September 2004 and 2005. In southern Belgium, we trapped in 9 sites once per season from summer 2004 to autumn 2005. To make the data from the two regions compatible, we took the average value for summer and autumn for the southern sites for the two years. We thus compared data from a non-epidemic year (2004) and an epidemic year (2005). In each study site, we constructed one 10 × 10 trapping grid of 100 live traps (Sherman Live Trap Co., Talahassee, FL), spaced at 10 meters intervals. Traps were set for three consecutive trap nights per site. Trapped bank voles were anaesthetised with Isoflurane (Forene, Abbott, Kent, UK), individually marked and released at their original place of capture after blood sample collection from the retro-orbital sinus. Plasma was obtained after immediate centrifugation and stored in liquid nitrogen in the field, and later at -80°C in the laboratory. A summary of field trapping data used is presented in Table 1.

**Table 1:** Summary of trapping results per field trapping site.

		Site number	Site name	Absolute number of bank voles	Number of bank voles tested	Number of PUUV positive bank voles	Prevalence (%)	CI for prevalence
**2004**	Northern Belgium	1	Groot Schietveld	32	20	0	0.00	0.00 – 16.68
		2	Driehoeksbos	8	7	0	0.00	0.00 – 37.71
		3	St. Jansberg	71	55	0	0.00	0.00 – 6.81
		4	Hustenveld	70	33	0	0.00	0.00 – 10.14
		5	Jongenbos	52	31	0	0.00	0.00 – 10.79
		6	Belle-Vue bos	87	59	0	0.00	0.00 – 6.35
		7	Alserbos	66	50	6	12.00	5.36 – 23.83
		8	Hornebos	71	43	1	2.33	0.12 – 12.37
	Southern Belgium	9	Anderlues	20	15	0	0.00	0.00 – 22.22
		10	Fontaine-l'Evêque	16	15	0	0.00	0.00 – 22.22
		11	Havelange	20	18	4	22.22	7.97 – 47.14
		12	Hamois	24	18	0	0.00	0.00 – 18.52
		13	Beaumont	152	137	34	24.82	18.18 – 32.80
		14	Somme-Leuze	90	69	5	7.25	2.90 – 15.78
		15	Chimay	21	21	0	0.00	0.00 – 15.89
		16	Viroinval	82	76	12	15.79	9.03 – 25.56
		17	Couvin	40	40	2	5.00	0.90 – 17.10
**2005**	Northern Belgium	1	Groot Schietveld	15	13	0	0.00	0.00 – 22.51
		2	Driehoeksbos	25	21	1	4.76	0.25 – 23.26
		3	St. Jansberg	46	38	1	2.63	0.14 – 14.00
		4	Hustenveld	15	9	0	0.00	0.00 – 32.33
		5	Jongenbos	4	2	0	0.00	0.00 – 77.63
		6	Bellevuebos	57	44	0	0.00	0.00 – 8.51
		7	Alserbos	47	39	6	15.38	6.92 – 30.54
		8	Hornebos	72	54	3	5.56	1.54 – 15.45
	Southern Belgium	9	Anderlues	46	39	0	0.00	0.00 – 8.59
		10	Fontaine l'Evêque	62	55	22	40.00	27.78 – 53.66
		11	Havelange	45	38	13	34.21	20.80 – 51.33
		12	Hamois	27	22	7	31.82	15.18 – 54.65
		13	Beaumont	84	77	34	44.16	33.03 – 55.87
		14	Somme-Leuze	97	85	5	5.88	2.35 – 13.33
		15	Chimay	145	129	28	21.71	15.32 – 29.78
		16	Viroinval	155	129	13	10.08	5.65 – 16.57
		17	Couvin	93	82	15	18.29	11.06 – 27.97

Number of trapped, tested and seropositive bank voles, and prevalence for each field trapping site. Site numbers refer to Figure 1. Data of southern Belgium correspond to the total number of bank voles captured in summer and autumn. Confidence intervals (CI) for prevalence were calculated using the Quantitative Parasitology free software [39].

##### Prevalence among bank voles

Collected plasma was screened by immunofluorescence assay, using spot slides with acetone fixed Vero E6 cells non-infected and infected with Puumala Sotkamo strain (HaartBio Ltd, Helsinki, Finland). Samples were diluted 1:10 and incubated on the spot slides, followed by incubation with 1:30 diluted FITC-conjugated polyclonal rabbit anti-mouse IgG (DakoCytomation, Glostrup, Denmark). Bank vole anti-PUUV monoclonal antibody 2E12 (HaartBio Ltd, Helsinki, Finland), dilution 1:500, was used as a positive control. Prevalence was calculated as the proportion of seropositive bank voles on the total number of individuals tested at a given point in time.

##### Abundance of bank voles

We used the absolute number of bank voles captured at each site as an indicator of the abundance of bank voles. This number does not include recaptures.

#### Landsat images processing

Environmental data were extracted from two Landsat-7 ETM+ images at 28.5 m resolution, acquired on 3 July 2001 (Path 198, Row 024, U.S. Geological Survey EROS (USGS), Sioux Falls; and Path 198, Row 025, Global Land Cover Facility (GLCF), Maryland). Images were geometrically and radiometrically corrected at the EROS Data Center of the USGS and georeferenced using 1:25,000 topographic maps provided by the Belgian National Geographical Institute (NGI). Using the standard supervised maximum likelihood method, we classified the image in order to obtain three land cover classes: deciduous forests, build-up areas, and other land covers. Training areas were determined based on topographic maps of Belgium (NGI). The accuracy assessment, based on a set of randomly distributed points with a minimum of 50 points in each land cover class, was performed using 1:25,000 topographic maps and field observations. Kappa classification accuracy statistics (KHAT) reached 0.85 in both cases with global accuracies of 0.94 and 0.93 [27].

##### Land-surface attributes

Four indices that characterize habitat quality were extracted from Landsat data. The Tasselled Cap transformation of Landsat ETM+ data provided three images clearly related to biophysical attributes: soil brightness (TCB), greenness of the vegetation (TCG), and soil and plant wetness (TCW) [28]. The Normalized difference vegetation index (NDVI) was also calculated using the near-Infrared (NIR) and red reflectance bands: NDVI=NIR−redNIR+red
 MathType@MTEF@5@5@+=feaafiart1ev1aaatCvAUfKttLearuWrP9MDH5MBPbIqV92AaeXatLxBI9gBaebbnrfifHhDYfgasaacPC6xNi=xH8viVGI8Gi=hEeeu0xXdbba9frFj0xb9qqpG0dXdb9aspeI8k8fiI+fsY=rqGqVepae9pg0db9vqaiVgFr0xfr=xfr=xc9adbaqaaeGacaGaaiaabeqaaeqabiWaaaGcbaGaemOta4KaemiraqKaemOvayLaemysaKKaeyypa0tcfa4aaSaaaeaacqWGobGtcqWGjbqscqWGsbGucqGHsislcqWGYbGCcqWGLbqzcqWGKbazaeaacqWGobGtcqWGjbqscqWGsbGucqGHRaWkcqWGYbGCcqWGLbqzcqWGKbazaaaaaa@42C8@. The NDVI can be interpreted in terms of fraction of photosynthetically active radiation absorbed by the vegetation canopy and canopy attributes (green biomass or green leaf area index). For each field site, a buffer of 50 meters was constructed around the site centroid. Most of these buffer areas are contained within the 100 × 100 meters trapping grids. The average values of TCB, TCG, TCW and NDVI within the buffer area of each field site were then extracted.

##### Landscape configuration

Field sites were located in deciduous forests of varying size, shape, and proximity to/connectivity with other forest patches. Based on the raster grid derived from Landsat imageries and composed of three discrete classes (deciduous forest, build-up areas and other), patches were delineated by aggregating adjacent cells that have the same value of land cover (considering the 8 neighbouring cells). We then computed local patch metrics for every discrete deciduous forest patch of the landscape, using the Fragstats 3.3 software [29]. We extracted the values of patch metrics obtained for the 17 forest patches where field trapping sites are located. Given the high correlations between patch metrics, we selected 4 metrics representative of the different categories available in Fragstats: (i) the area, (ii) the contiguity index, which assesses the spatial connectedness of cells within a patch, (iii) the core area index, which represents the percentage of the patch considered as 'core area', with an edge depth of 100 meters, and (iv) the proximity index, which considers the size and proximity of all forested patches whose edges are within a distance of 1000 meters from the focal patch. The metrics were selected based on the statistical significance of Pearson correlation coefficients with the absolute number of bank voles captured and PUUV prevalence among bank voles, and their ease of interpretation. In addition to forest patch configuration, surrounding land cover types (i.e., the land cover matrix around forest patches) are likely to influence the connectivity between patches and thus rodent's abundance and prevalence [5]. We calculated the proportion of build-up areas in buffers of 2 kilometres around forest patches, which can be considered as barriers for rodent's movements.

#### Soil data

The "Aardewerk" database, composed of more than 13,000 geo-referenced and analysed soil profiles distributed all over Belgium, provides extensive and accurate data on soil characteristics. Data were collected during the 1950–1970 period and later digitized [30,31]. 17 points from the Aardewerk database were selected and allocated to each field trapping site based on: (i) membership of the same soil association (based on the soil map of Belgium from [32]) and (ii) minimum distance to field sites. Every field site belongs to the same soil association as its allocated Aardewerk point, except for two sites (Fontaine-l'Evêque and Groot Schietveld) for which we took the closest point independent from soil association, given the absence of nearby points from the same association. The maximum distance between soil data points and field sites is 6.7 km (with an average of 1.75 km), which is below the range of variation of soil texture at the level of the country. We extracted the proportion of thin particles (smaller than 10 μm) in the upper horizon of soil profiles. The proportion of thin particles was used as a proxy for soil moisture. For the 8 sites in Wallonia, field measurements of soil moisture were collected. These empirical data were correlated to the data from the Aardewerk database used here (r = 0.63 and p = 0.07).

#### Climate data

The Royal Meteorological Institute of Belgium (RMI, Uccle) provided daily data on air temperature and precipitation from 2003 to 2006 throughout Belgium (minimum, maximum and average temperatures for 176 stations, and precipitation for 272 stations). We calculated seasonal averages of minimum, maximum and average temperatures and precipitation. Stations were assigned to field sites based on minimum distance criteria. For precipitation, the maximum distance between selected stations and field sites is 8.6 km (with an average of less than 4.5 km). This distance is below the range of spatial variability of seasonal averages of precipitations in temperate regions.

#### Number of NE cases among humans

Data on human NE cases between 1994 and 2005 – i.e., the number of new cases of NE diagnosed during this period – were provided by the Scientific Institute of Public Health (IPH, Brussels) per postal code (a spatial entity smaller than the municipality). There is no mandatory registration of NE in Belgium, but sentinel laboratories send positive samples to Reference Laboratories for confirmation, which then submit monthly data on confirmed cases to the IPH. Duplicate records were eliminated.

### Statistical analyses

First, we investigated changes in bank vole abundance (estimated by the absolute number of bank voles captured) and PUUV prevalence between 2004 and 2005. We then performed multivariable regressions in order to identify environmental variables that influence the abundance and prevalence of bank voles in Belgian broad-leaf forests. For each regression, the independent variables were related to land-surface attributes (TCB, TCG, TCW, and NDVI), landscape configuration (patch area, patch contiguity, core area index, proximity, and the proportion of build-up areas around the forest patch), soil (proportion of thin particles), climate (seasonal temperatures and rainfall), and the absolute number of bank voles trapped for the model on prevalence. Logarithmic transformations were used for two of the landscape configuration variables (patch area and proximity) to improve the linearity of the data. All other variables were normally distributed. We first performed linear regressions with the absolute number of bank voles trapped as dependent variable. We carried out separate linear regressions for the two years, given their epidemic (2005) and non-epidemic (2004) status. This reduces the number of observations for statistical analysis but increasing the number of trapping sites would come at a high cost in terms of fieldwork.

To analyse the prevalence data, we then used a logistic model with logit link function and binomial distribution. For each of the 17 sites, the proportion number of seropositive bank voles/number of bank voles tested, was used as dependent variable. In addition to the significance of variables, the adjusted-R^2 ^for the linear regressions and the ratio between the residual deviance and the number of degrees of freedom for the logistic regressions were used to select variables to retain in the final regression models. Variables were tested for collinearity and strongly related variables were not introduced in the same model (e.g., TCG and NDVI).

To extend the study to the PUUV transmission risk to humans, we also analysed the relationships between the above environmental variables and the total number of NE human cases diagnosed between 1994 and 2005. Analyses were performed on the 17 postal code areas where field sites were located. We extracted the average value of variables related to land-surface attributes for forest patches that were at least partially included in the postal code areas. Given the overdispersed (i.e. variance higher than mean) distribution of human cases, we performed a negative binomial regression, with the number of NE cases per postal code area as dependent variable. The population number of postal codes was added as offset variable in the model. All statistical analyses were performed using SAS^® ^v. 9.1 [33] or the R statistical software [34].

## Results

### Abundance of bank voles and PUUV prevalence

**Figure 2:**
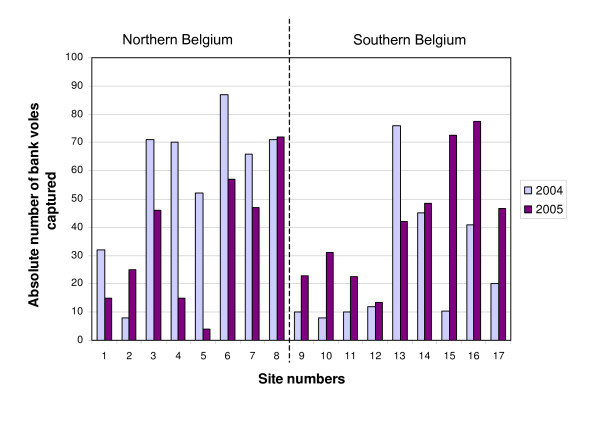
**Absolute number of bank voles per ha captured in 2004 and 2005 for each field trapping site**. Field sites are classified from high latitudes (northern Belgium) to low latitudes (southern Belgium). Site numbers refer to Figure 1.

**Figure 3:**
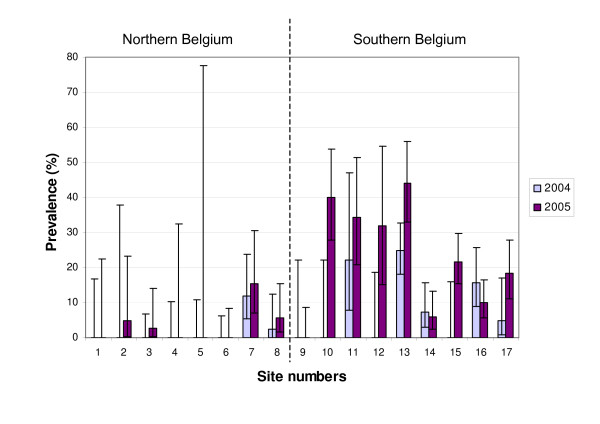
**PUUV prevalence for 2004 and 2005 for each field trapping site**. Field sites are classified from high latitudes (northern Belgium) to low latitudes (southern Belgium). Site numbers refer to Figure 1. Confidence intervals were calculated using the Quantitative Parasitology free software [39].

The average number of bank voles trapped was 41 in 2004 and 39 in 2005. The abundance of bank voles was thus stable. However, abundance was higher in northern Belgium in 2004 and lower in 2005 compared to southern Belgium (Table 2, Figure 2). There were thus changes in bank vole population dynamics between the two years. The average prevalence significantly increased from 2004 (5.56% per site) to 2005 (12.30% per site) (Wilcoxon non-parametric test significant at p = 0.004) (Figure 3). 7 sites out of 17 (41%) were infected in 2004 compared to 12 sites out of 17 (71%) in 2005. No linear relationship was observed between the abundance of bank voles and PUUV prevalence, neither for 2004, nor for 2005. This confirms that a positive correlation between host abundance and prevalence is rarely observed at a seasonal time scale [35]. However, when combined with environmental data, the abundance of bank voles appeared as a significant explanatory factor in the model on prevalence in 2004 (Table 3).

**Table 2:** Abundance and prevalence of bank voles per region

Year	Region	Average number of bank voles	Average prevalence (%)
2004	Northern Belgium	57	1.79
	Southern Belgium	26	9.33
2005	Northern Belgium	35	3.54
	Southern Belgium	42	20.08

Average values for the number of bank voles trapped and the PUUV prevalence in bank voles for the northern and southern parts of Belgium during a non-epidemic year (2004) and an epidemic year (2005).

Multivariable regressions on abundance and prevalence of bank voles show different results for 2004 and 2005 (Table 3). The abundance of bank voles in 2004 is explained by variables related to land-surface attributes, landscape configuration and climate, with an adjusted-R^2 ^of 0.64. The explanatory power of the model on the abundance of bank voles for 2005 is weaker (adjusted-R^2 ^of 0.30) and variables are only related to the landscape configuration and climate conditions. For PUUV prevalence, significant variables are only related to climate conditions and the abundance of bank voles. These relationships are discussed below.

**Table 3:** Results from regression models.

Statistical model	Linear regression		Logistic regression		Negative binomial regression
Dependent variable	**Number of bank voles**		**PUUV prevalence**		**Number of human NE cases**

	2004	2005	2004	2005	1994–2005
**Land-surface attributes**					
Greenness of the vegetation	1.75**	-	-	-	-
**Landscape configuration**					
Area of forest patch	-	-	-	-	0.02*
Proximity index	-6.39**	-	-	-	-
Proportion of build-up areas around the forest patch	-	-145.73*	-	-	-
**Soil**					
Proportion of thin particles (< 10 μm)	-	-	-	-	0.20***
**Climate**					
Maximum temperature during the previous winter	-	-	-0.22***	-0.19***	-
Rainfall during the previous autumn	-	-	-	-0.01*	-
Rainfall during the previous winter	-0.30*	-	-	-	-
Rainfall during the previous spring	-	-0.37*	-	-	-
**Absolute number of bank voles**	-	-	0.02*	-	-
					
**Goodness of fit**					
N	17	17	17	17	17
Events/Trials	-	-	35.5/502.5	79.5/548	-
R^2^	0.71	0.39	-	-	-
Adj. R^2^	0.64	0.30	-	-	-
Degrees of freedom	-	-	14	14	14
Residual deviance	-	-	14.47	20.67	10.51
Deviance value/DF	-	-	1.03	1.48	0.75

Parameter estimates of significant variables and goodness of fit statistics for: (i) linear regressions on the absolute number of bank voles captured, (ii) logistic regression with logit link function and binomial distribution on the PUUV prevalence of bank voles, and (iii) negative binomial regression on NE cases per postal code area. For southern Belgium, data used in statistical analyses correspond to the average number of bank voles captured in summer and autumn. The dispersion parameter for the negative binomial family was taken to be 1. *** P-value < 0.001; ** P-value < 0.01; * P-value < 0.1.

### Land-surface attributes and landscape configuration

As expected, vegetation greenness, which is linked to habitat quality and food availability for rodents, was positively related to the absolute number of bank voles captured, but only in 2004. In 2005, this variable was not related to the absolute number of bank voles captured (Pearson correlation coefficient r = 0.19; p = 0.46). Two variables that represent characteristics of the landscape configuration were significant. Both are proximity metrics. The proximity index represents the size and proximity of forest patches that are in a search radius of 1,000 meters from the focal patch. The proportion of build-up areas around forest patches also influences the proximity between forest patches by impeding the movements of bank voles. For the non-epidemic year, in 2004, the number of bank voles captured was higher in more isolated forest patches. However, in 2005, this number was higher in forest patches with a low proportion of build-up areas around them, i.e. in less isolated patches.

### Soil

We expected a higher prevalence where the proportion of thin particles (i.e. clay and thin loam) is higher, i.e. where soil moisture remains higher. Other studies observed that soil moisture favours the persistence of the virus in soil litter [3,17]. However, soil variables were not significant in the models on abundance and prevalence, probably because the proportion of thin particles follows a North-South gradient similar to the gradient of air temperature, leading to a high correlation between these two variables for the trapping sites (r = -0.64; p = 0.006). In the southern Belgium sites, winter temperatures are low and the clay fraction is generally high compared to the northern Belgium sites.

### Climate

PUUV prevalence is strongly associated with maximal temperatures from the previous winter. This supports the hypothesis that low maximal winter temperatures increase the survival of the virus [4]. The amount of precipitation during the previous autumn influences negatively the prevalence in 2005. Climate conditions also influence the absolute number of bank voles captured: bank voles are negatively influenced by high winter and spring precipitations.

### Human cases

The negative binomial regression model on human NE cases per postal code area has two significant variables: the area of forest patches in the postal code area and the proportion of thin particles in soils, both with a positive sign (Table 3). The size of soil particles influences the transmission of the virus to humans, by favouring the indirect transmission. Actually, the virus persists in soil litter for longer where soil moisture is higher [3,17].

## Discussion

The environmental determinants of the spatial distribution of bank voles vary between the two years studied here. Between 2004 and 2005, we observed a general increase in the abundance of bank voles in southern compared to northern Belgium. The abundance of bank voles in 2004 is better explained by environmental variables than in 2005, with an adjusted-R^2 ^more than twice as high. This suggests that the spatial behaviour of bank voles is different during epidemic and non-epidemic years. During non-epidemic years (2004), bank voles are found in high-quality (high vegetation greenness values) and isolated forest patches. Bank voles living in high-quality patches are thought to better survive during all seasons of non-epidemic years than bank voles living in low-quality patches, as was shown for the bank vole [8] and the Mediterranean pine vole [36]. During the epidemic year (2005), the abundance was higher in less isolated but lower quality patches, suggesting that there was a high dispersal of bank voles between these two years. This could be due to an increase in bank vole abundance in southern Belgium during the epidemic year, leading to a spread to less suitable patches. Well-connected patches have more chances to be recolonized after local extinctions [7]. This is consistent with the first part of our hypothesis, i.e. that landscape attributes influence the abundance of hosts.

The prevalence among bank voles depends largely on climate conditions, particularly temperature during the previous winter. The number of NE cases among humans depends on soil attributes. This is consistent with the second part of our hypothesis, i.e. that climate and soil conditions influence the persistence of PUUV in the environment and thus the indirect transmission to bank voles and humans. Our results suggest that low winter temperatures and high soil moisture favour the survival of the virus.

In addition to climate conditions, PUUV prevalence among bank voles depends on the abundance of bank voles. During the non-epidemic year, a high number of bank voles is associated with a high prevalence, suggesting that a critical number of bank voles is required for infection to subsist.

Note that the landscape structure could also influence the virus occurrence in hosts by controlling movements of individuals and thus contact rate between infected and susceptible rodents. Langlois et al. [20] observed that the hantavirus incidence in deer mice was higher in landscapes with a higher level of fragmentation of the preferred habitat. This effect was not visible in our models.

Disease risk thus depends on a combination of various environmental factors. To understand the causal links between the environment and NE transmission risk, it is important to distinguish between environmental factors related to the abundance of hosts (land-surface attributes, landscape configuration and climate), as a function of host ecology, and environmental factors related to PUUV prevalence (mainly winter temperatures and soil moisture), as a function of virus ecology. For indirectly-transmitted zoonoses, the impact of the environment on disease risk cannot be reduced to its impact on the spatial distribution of hosts. Within the temporal scale of our study, the size of the host population influences the prevalence among bank voles, but in opposite directions depending on the epidemic or non-epidemic character of the year. The presence of a population of rodents is necessary for PUUV transmission to humans, but it is not a sufficient condition, nor does an increase in the population size necessarily lead to an immediate increase in transmission risk. Prevalence among bank voles and the presence of environmental attributes favourable to the indirect transmission seem to be more important factors. For example, northern Belgium has high densities of rodents but a low PUUV prevalence, more sandy soils, and warmer winter temperatures compared to southern Belgium, and thus few NE human cases [37]. Moreover, the relationship between environmental factors and bank vole's abundance was not consistent for the two years considered and thus has a low predictive power of bank vole abundance within broadleaf forest patches.

A high PUUV prevalence among rodents appears to be an essential factor for the transmission to humans. Our results suggest that viral prevalence in rodents is largely influenced by the efficiency of the indirect transmission path, which makes the transmission rate less dependent on the host abundance. Human contamination seems also to occur principally by this path [3]. This was confirmed by our analysis on NE cases. The number of NE cases is strongly related to the proportion of thin particles in the soil, which favours the indirect transmission path via soil moisture.

However, even if soil types follow a North-South gradient similar to the gradient of prevalence in Belgium, this does not necessary imply causality between these two factors as other environmental factors follow the same spatial gradient. Elevation is higher in southern Belgium and temperatures are consequently lower in this region. Fine scale, experimental studies should be conducted to confirm the link between PUUV prevalence and soil types. The relationship between soil moisture (estimated via the proportion of thin particles in the soil in our study) and PUUV longevity outside the host has been demonstrated based on observations within an enclosed bank vole system [17], theoretical modelling [3] and laboratory experiences [4].

The area of forest patches is a good explanatory factor for the number of human cases. It could potentially have three impacts on the transmission of a non-vector-borne zoonotic disease: on the abundance of bank voles by providing a suitable habitat, on the contact rate between bank vole populations and thus on virus transmission among bank voles, and/or on human risk behaviours related to an increase in forest-related activities in regions with a large forest cover. The area of forest patches was not significant in models explaining the abundance and prevalence of bank voles. Human risk behaviours, such as the propensity to go in forests, are likely to have an important impact on disease risk among humans [38]. The transmission risk to humans seems therefore to depend also on the contact rate between humans and infected environments [38]. The likelihood for people going in forests is expected to be higher in areas with large forests. As northern Belgium is characterized by small forest patches, human activities in forests are less important and the transmission risk is expected to be lower in this region, thus leading to few human cases. To sum up, in Belgium's broadleaf forests, when bank vole numbers are high, climatic and soil factors favouring the persistence of the virus and forest-related land uses are likely to better predict the disease risk for humans than simply the abundance of hosts.

## Conclusion

Spatial associations between environmental conditions and the incidence of non-vector-borne zoonoses may involve complex causal pathways. Beyond a threshold abundance of hosts, environmental factors influencing the prevalence of the infection in the host can better predict the number of cases of non-vector-borne diseases among humans than environmental factors associated with host habitats that influence the abundance of hosts. The indirect transmission path of PUUV seems to be important in Belgian forests, particularly in the southern region, as the prevalence of bank voles and the number of human cases are mainly influenced by winter temperatures and soil characteristics that control the persistence of the virus in the environment. The landscape configuration influences host populations but also human risk behaviour, and particularly the propensity for people to go in areas that best support the virus.

## Competing interests

The author(s) declare that they have no competing interests.

## Authors' contributions

CL carried out the remote sensing data processing, extracted environmental data, performed the statistical analyses, interpreted the results, and wrote the first draft of the manuscript. KT collected data on the abundance and prevalence of bank voles, carried out the field and laboratory work, and helped in the design of the study and the interpretation of results. HL helped in the design of the study and the interpretation of results. EFL led the design of the study, advised CL, and contributed to writing the manuscript. All authors read and approved the final manuscript.
